# Copper-Mediated Homocoupling of *N*-propargylcytisine—Synthesis and Spectral Characterization of Novel Cytisine-Based Diyne Dimer

**DOI:** 10.3390/molecules30193955

**Published:** 2025-10-01

**Authors:** Anna K. Przybył, Adam Huczyński, Ewa Krystkowiak

**Affiliations:** 1 Department of Medical Chemistry, Faculty of Chemistry, Adam Mickiewicz University, Uniwersytetu Poznańskiego 8, 61-614 Poznań, Poland; 2 Department of Spectroscopy and Magnetism, Faculty of Chemistry, Adam Mickiewicz University, Uniwersytetu Poznańskiego 8, 61-614 Poznań, Poland

**Keywords:** *N*-heterocycles, alkaloids, (−)-cytisine derivatives, *N*-propargylcytisine, Glaser-Hay coupling, 1,3-diynes, alkaloid functionalization, UV-VIS absorption spectroscopy

## Abstract

Cytisine, a naturally occurring alkaloid and partial agonist of nicotinic acetylcholine receptors (nAChRs), has long been used as a smoking cessation aid and serves as the pharmacophore for varenicline. Recent research has expanded its therapeutic scope to neurodegenerative and neurological disorders, motivating the development of new cytisine derivatives. Among these, *N*-propargylcytisine combines the biological activity of the parent compound with the synthetic versatility of the terminal alkyne group. Herein, we report the synthesis and characterization of *N*-propargylcytisine, and its symmetrical dimer linked through 1,3-diyne moiety obtained via a copper-mediated Glaser–Hay oxidative coupling. The products were analyzed by NMR, FT-IR, and mass spectrometry, confirming the introduction of the propargyl moiety and the formation of the diyne bridge. Solvatochromic study of both compounds were performed using UV-VIS absorption spectroscopy in solvents of varying polarity, including protic solvents capable of hydrogen bonding. The 1,3-diyne motif, commonly found in bioactive natural products, endows the resulting dimer with potential for further derivatization and biological evaluation. This study demonstrates the utility of the Glaser–Hay reaction in the functionalization of alkaloid scaffolds and highlights the prospects of *N*-propargylcytisine derivatives in drug discovery targeting the central nervous system.

## 1. Introduction

**Figure 1:**
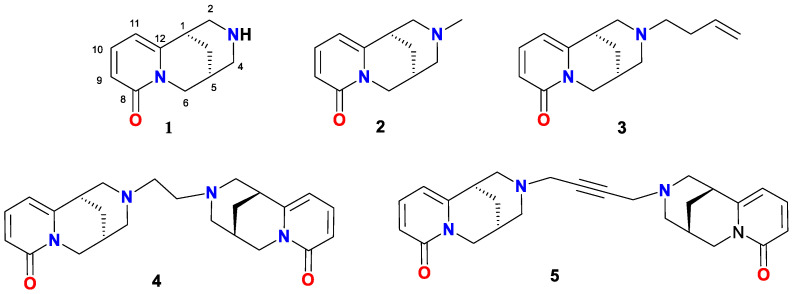
(−)-Cytisine (**1**) and its derivatives **2–5**.

(−)-Cytisine (**1**, Figure 1), a naturally occurring quinolizidine alkaloid, has intrigued scientists and medical professionals for over a century due to its unique pharmacological properties and therapeutic potential. Initially isolated from *Cytisus laburnum* seeds [1,2], cytisine is predominantly found in plants of the Leguminosae family, such as *Laburnum*, *Thermopsis*, *Cytisus*, *Genista*, and *Sophora* genera [1,2,3]. Its natural (−)-enantiomer exhibits a remarkable affinity for nicotinic acetylcholine receptors (nAChRs), a feature that underpins its pharmacological relevance, in contrast to its synthetic (+)-enantiomer that demonstrates significantly weaker interactions with these receptors [1,4].

This receptor-binding capability places cytisine among the notable modulators of the cholinergic system. Acting as a partial agonist of nAChRs, cytisine not only mimics nicotine’s action but also interferes with its addictive potential by modulating dopaminergic transmission and reducing the reinforcing effects of nicotine [1,5,6,7,8]. These properties have made cytisine an effective aid in smoking cessation [1,5,9,10]. Its potential neuroprotective effects, including its ability to mitigate neurodegenerative conditions like Parkinson’s disease, by modulating dopamine release and reducing oxidative stress in neuronal tissues, have been highlighted [3,11,12]. Additionally, its antiepileptic properties, observed through the activation of α7 nAChRs, offer promising avenues for conditions such as temporal lobe epilepsy [9,12].

Moreover, the structural versatility of cytisine has inspired the development of numerous derivatives with enhanced or diversified pharmacological profiles. By introducing modifications at specific positions or substituting the nitrogen atom, its potential applications across various therapeutic domains have been expanded [13,14,15,16,17]. For instance, *N*-substituted derivatives like *N*-methylcytisine (**2**, Figure 1) and *N*-butenylcytisine (**3**, Figure 1) have been investigated for their interactions with nAChRs [18,19], while dimeric compounds like 1,2-bis*N*-cytisinylethane (**4**, Figure 1) have demonstrated selective efficacy in modulating nicotine addiction behaviors in vitro and in vivo [6]. Additionally, 1,4-bis*N*-cytisinylbutyne (**5,** Figure 1), has been shown to exhibit nicotine-like reinforcing properties and the ability to block nicotine-induced conditioned place preference when co-administered with nicotine, suggesting its potential for smoking cessation therapies [18,19].

While cytisine is well-established as a therapeutic agent in nicotine dependence, its potential applications beyond tobacco addiction have been recently revealed [1,10,20,21]. Beyond its well-documented role in nicotine dependence, cytisine is emerging as a compound of interest in broader therapeutic contexts. In particular, cytisine (1) has gained renewed attention also because of its ability to reduce various ethanol-related behaviors and ethanol-induced striatal dopamine activity [22,23,24]. Furthermore, cytisine (1) has been investigated for its cardiovascular effects [25]. Efforts have also been directed toward improving its pharmacokinetic properties, such as enhancing blood–brain barrier permeability through increasing lipophilicity [26], although even relatively simple modifications, such as the formation of cytisine salts, have attracted considerable research interest [27]. Moreover, certain *N*-benzylcytisine derivatives have demonstrated significant antiproliferative activity against human cancer cell lines, while displaying reduced toxicity towards normal murine fibroblasts in comparison with the commonly used chemotherapeutic agent—cisplatin [28].

**Figure 2:**
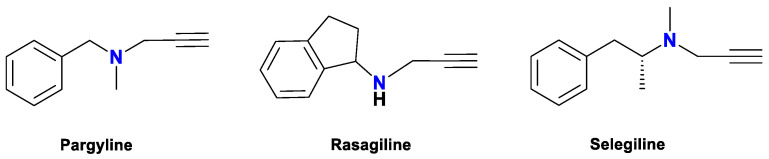
Derivatives of propargylamine against neurodegenerative diseases [29].

In recent years, the propargylamine motif’s wide utility in drug development has prompted studies on the incorporation of propargyl groups into the cytisine scaffold. Propargyl-containing compounds, including rasagiline, selegiline, and pargyline (Figure 2), are used clinically to treat Parkinson’s and Alzheimer’s diseases due to their neuroprotective properties and selective monoamine oxidase inhibition [29]. Moreover, from a synthetic chemistry standpoint, the terminal alkyne present in propargylamine serves as a reactive handle for numerous transformations, owing to its capacity to undergo nucleophilic and electrophilic reactions, as well as participate in metal-catalyzed coupling strategies [29].

### 1.1. Glaser Coupling Reaction and Its Developments

**Scheme 1:**
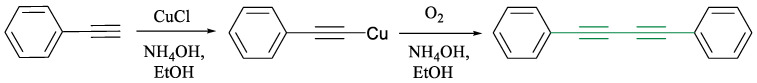
Phenylacetylene’s dimerization using the Glaser coupling reaction [30].

**Scheme 2:**
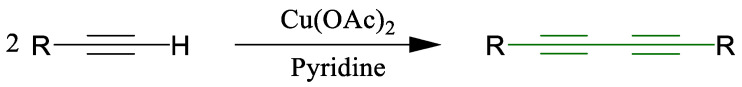
Synthesis of diynes developed by Eglinton and Galbraith [31].

One of the most versatile transformations involving terminal alkynes is the copper-mediated oxidative coupling known as the Glaser reaction. First reported in 1869 by Carl Glaser for the formation of symmetrical 1,3-diynes (Scheme 1) [30], this reaction was initially limited by the instability of aliphatic copper acetylide intermediates. A significant improvement was made by Eglinton and Galbraith in the 1950s through the use of copper(II) acetate in pyridine and methanol, enhancing efficiency (Scheme 2) [31].

**Scheme 3:**
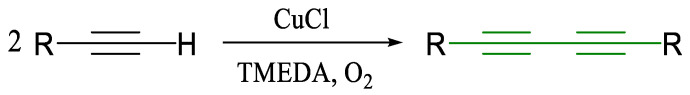
Modification of the Glaser coupling reaction by Hay [32].

A breakthrough came in 1962 with Allan Hay’s introduction of the Glaser–Hay protocol, which makes use of CuCl and TMEDA under aerobic conditions (Scheme 3) [32].

**Scheme 4:**
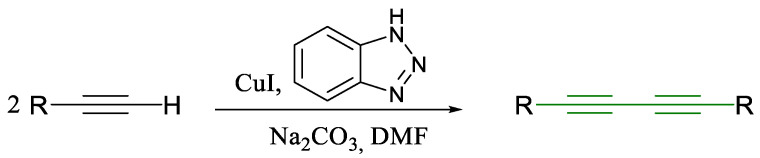
Glaser coupling reaction using benzotriazole as a ligand [34].

**Scheme 5:**
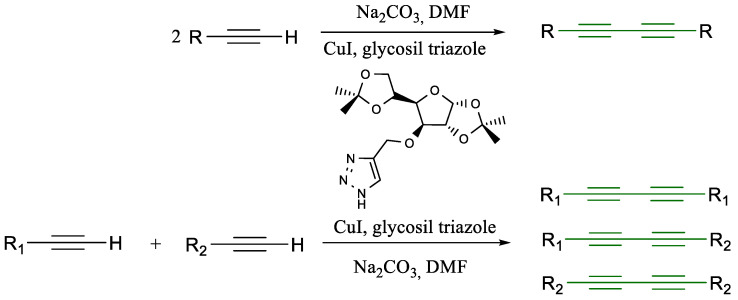
Glaser coupling reaction with glycosyl triazole [35].

**Scheme 6:**
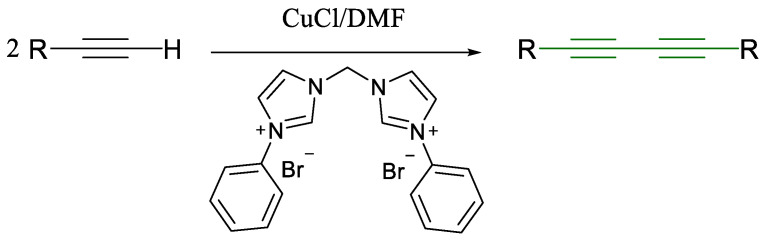
Glaser coupling reaction with bis-*N*-heterocyclic carbine [36].

This variant increased solubility and reactivity, leading to more reliable and high-yielding outcomes. Building on these developments, various copper systems have since emerged, e.g., CuI/NBS/DIPEA [33], CuI/benzotriazole [34], CuI/glycosyl triazoles [35], and CuCl with bis-NHC ligands [36], offering high efficiency, substrate tolerance, and compatibility with milder or solvent-free conditions (Scheme 4, Scheme 5 and Scheme 6) [33,34,35,36].

**Scheme 7:**
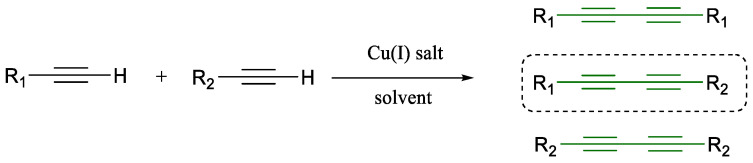
Glaser coupling’s chemo-selectivity issues [37].

However, a persistent limitation remains: the lack of chemo-selectivity in cross-coupling two distinct alkynes. The resulting statistical mixture of symmetrical and unsymmetrical diynes poses a purification challenge (Scheme 7) [37]. Although strategies such as polarity-based separation and solid-supported reactions have been explored, the problem remains largely unsolved.

**Scheme 8:**
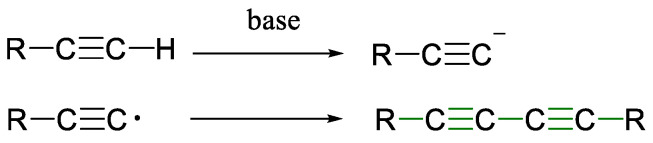
Formation of diynes via Glaser coupling reaction [30].

**Scheme 9:**
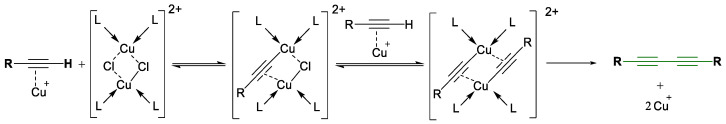
Glaser coupling reaction’s mechanism proposed by Bohlman [38].

Mechanistic studies have elucidated various pathways, from radical intermediates to dicopper complexes, but recent proposals suggest that a Cu–alkynyl intermediate undergoes oxidation by molecular oxygen to form the diyne product (Scheme 8 and Scheme 9) [36,37,38]. These findings continue to shape the design of new catalysts and protocols.

**Figure 3:**
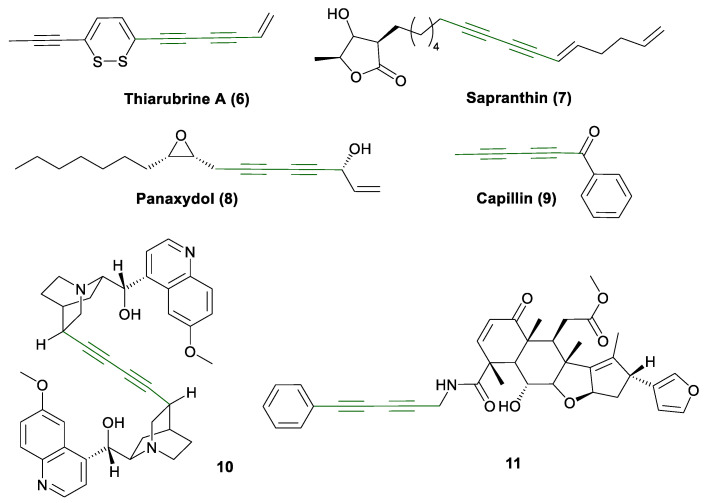
Bioactive 1,3-diynes **6–11** [43,44,46].

The 1,3-diyne motif is widely encountered in nature and medicinal chemistry, and the syntheses of such diynes have been widely described, especially symmetrical ones [39,40,41]. The renewed interest in unsymmetrical compounds arises not only from their abundance in nature but also from their structural complexity, which allows for diverse functionalization. Unsymmetrical diynes are particularly notable for their biological activity and chemical stability [42], and they occur in a broad range of organisms including plants, fungi, and marine species [43,44]. The range of their biological activity includes antibacterial (**6**), antimicrobial, antifungal (**7**), antitumor and anticancer (**8** and **9**, Figure 3), anti-HIV, and pesticidal properties [43,44]. In the synthetic contexts, diynes have been incorporated into alkaloid dimers and complex bioactive frameworks, such as quinidine derivatives (**10**, Figure 3) [45] and nimbolide-inspired anticancer agents (**11**, Figure 3) [46].

**Scheme 10:**
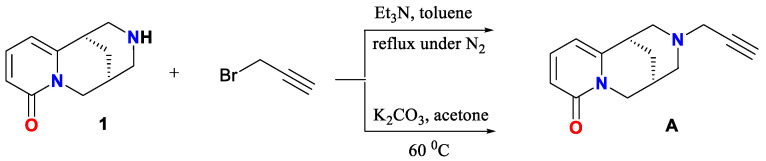
Synthesis of **A** by Nicolotti [18] and by Nurkenov [47].

Building on previous findings, the design and synthesis of *N*-propargylcytisine (**A**, Scheme 10) offer a promising strategy to combine the neuroactive properties of cytisine with the synthetic versatility provided by an alkyne moiety.

Notably, previous reports on the preparation and characterization of this derivative served as an important source of inspiration for our work, motivating us to pursue structural modification of the title compound using the Glaser reaction. *N*-propargylcytisine (**A**) has been synthesized by two independent groups through *N*-alkylation of cytisine with propargyl bromide under base conditions, either in refluxing toluene under nitrogen atmosphere (yielding 60%) [18] or in acetone at 50 °C (yielding 65%) [47]. While Nicolotti et al. focused primarily on the receptor affinity (pKi = 7.00) and SAR evaluation using CoMFA, Nurkenov et al. emphasized structural characterization using X-ray crystallography [18].

**Scheme 11:**
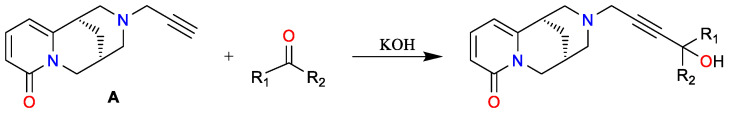
Synthesis of aminoacetylenic alcohols [47].

**Scheme 12:**
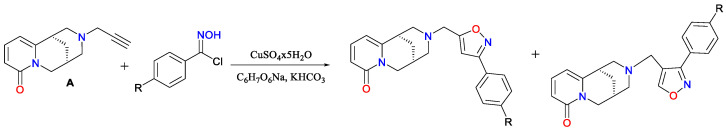
Synthesis of isomeric 3,5- and 3,4-disubstituted isoxazoles [48].

Furthermore, this derivative (**A**) has been used as a key intermediate in subsequent synthetic efforts, leading to the formation of aminoacetylenic alcohols (Scheme 11) and triazole-linked derivatives (Scheme 12) via copper(I)-catalyzed 1,3-dipolar cycloadditions with *N*-hydroxybenzene-carboximidoyl chlorides [48]. These compounds are under investigation for potential biological activity, particularly in relation to nAChR modulation and neuropharmacological applications. Another motivation for employing copper salts was the work of Pérez [49], who achieved *N*-arylation of cytisine under mild conditions via copper-catalyzed Chan–Lam coupling, enabling the synthesis of novel chemical entities and expanding the structural diversity of cytisinoids.

In light of these findings, the development of cytisine-based derivatives bearing propargyl functionalities and their subsequent transformation through Glaser-type oxidative coupling offers a promising pathway toward novel bioactive compounds; however, we have undertaken the synthesis of 2,4-diyne molecules with potential roles in neurodegenerative disease treatment and addiction therapy. Furthermore, the experimental design undertaken to determine whether the intrinsic copper-binding capacity of cytisine [50], would interfere with, or exert any modulatory effect on, the Glaser–Hay oxidative coupling of *N*-propargylcytisine, has indicated that such a coordination might even preclude the reaction from proceeding altogether.

### 1.2. Solvatochromic Study

Recognition of the effects of (−)-cytisine and its derivatives in medical therapies and their significance in the treatment of neurogenerative diseases and addiction therapy, indicated the need to understand the mode of their activity in biological systems. The initial stage of this type of research is to resolve the structure and specificity of the interaction of the compound studied in a less complex environment, e.g., in single- or two-component solvents of different properties, such as polarity or hydrogen-bond formation ability [51,52]. The UV-VIS absorption spectroscopy allows for obtaining information on the influence of the solvent properties on the type and energy of solute–solvent interactions in the ground S_0_ and in electronically excited states S_n_, n ≥ 1, which can be crucial for understanding the interactions of newly synthesized compounds in more complex systems.

The solute–solvent interactions are generally divided into two types: nonspecific, which are caused by polarity and polarizability, and specific such as donor–acceptor interactions, acid–base interactions, including hydrogen bonds, charge–transfer (CT) interactions, and π-π electron interactions. That is why proper interpretation and description of the relation between spectral properties of a solute and the properties of the solvent require prior determination of the solvent polarity and polarizability and its capability of hydrogen bond formation [53].

Nonspecific interactions can be well approximated by electrostatic models used in the theory of electrical conductivity of liquid media. Onsager’s electrical percolation theory assumes that the solute molecule occupies a cavity in a dielectric with relative permittivity ε and a refractive index n. It is assumed that the dominant effect is the interaction of the dipole moment with the reaction field generated in the spherical cavity because of the polarization of the medium by this dipole. In the expressions describing the energy of solute–solvent interactions, used in solvatochromic studies, the solvent is approximated as a continuous medium using the solvent polarity function, also called the solvatochromic function. The most common definition is the one given by Lipert and Mataga [54]:

(1) $$f\left(\varepsilon ,n^{2}\right)\;=\;\frac{\varepsilon -1}{2\varepsilon +1}-\frac{n^{2}}{2n^{2}+1}$$

The classification of solvents according to polarity and hydrogen bond formation ability usually uses the criteria based on spectral absorption measurements, proposed by Kamlet and Taft [55] and Catalan [56]. The solvents showing the ability of hydrogen bond formation are acceptors or donors of hydrogen bonds. The acceptor properties of the solvent depend on its ability to accept a hydrogen atom from the solute in order to form a hydrogen bond (β in Kamlet-Taft scale and SB in Catalan scale), while its donor properties depend on its ability to donate a hydrogen atom in order to form a hydrogen bond with the solute (α in Kamlet-Taft scale and SA in Catalan scale). The scale of α values extend from 0.0 for the solvents not able to donate hydrogen atoms to 1.96 for 1,1,1,3,3,3-hexafluoroisopropanol, HFIP, while the scale of β extends from 0.0 for the solvents whose molecule has no heteroatoms with a lone electron pair to 1.0 for triamide of hexamethylphosphoric acid, HMPA [57]. The properties of the selected solvents are collected in Table 1.

The term solvatochromic shift refers to the shift in absorption spectra of the solute observed in different solvents. Changes in the position of absorption band upon changing the solvent are due to the differences in the interaction of the solute molecules in the ground state S_0_ and in the electronically excited state (exemplary S_1_) with the solvent. Bands in absorption spectra can shift towards shorter wavelengths (hypsochromic shift, blue shift) or longer wavelengths (bathochromic shift, red shift).

Changes in the spectral behavior of a solute upon changing the solvent are consequences of the specific (hydrogen bonds) and nonspecific interactions between the solute and solvent molecules. If the solute molecules contain donor and/or acceptor groups, correct interpretation of spectral data needs separation and independent determination of these two types of interactions. Therefore, the vital aspect of such studies is the proper choice of solvents, either those that interact only nonspecifically with the solute or those that can form hydrogen bonds of a specific type [58].

Results of the absorption and emission spectral studies of (−)-cytisine in solvents of different polarities have shown that (−)-cytisine in n-hexane and acetonitrile, in the ground S_0_ and excited state S_1_, exists in the form of two chair conformers [59,60]. The fluorescence of both conformers of cytisine in n-hexane (a non-polar solvent) occurs from the excited state S_1_(n,π*) [59], while in polar acetonitrile, both conformers emit from the S_1_(π,π*) state [60].

The UV-VIS absorption spectra of *N*-methylcytisine, a quinolizidine alkaloid derivative of cytisine revealed absorption bands at 231 and 306 nm and a weak band at 295 nm in aqueous solution, well corresponding to the spectrum calculated at the B3LYP/6-311++G** basis set in water. The calculation predicted the same absorption spectra for the two possible conformers of *N*-methylcytisine, and the bands observed are evidently associated with π→π* and n→π* transitions [61]. Similar in shape UV-VIS absorption spectra with a long wavelength band maximum about 303 nm of cytisine and *N*-methylcytisine in methanol were recorded on a HPLC system with diode array detection [62,63].

Spectral characterization of several 3-amino derivatives of *N*-methylcytisine in water revealed differences in the shape of absorption and emission spectra. However, the positions of the long wavelength band maximum in their spectra were not significantly different. The absorption maximum was at about 320 nm and the fluorescence band appeared at about 402–405 nm, giving a Stokes shift of about 6400–6500 cm^−1^. The spectral parameters: molar absorption coefficient at the long wavelength band maximum in the range of 800–900 M^−1^cm^−1^, fluorescence quantum yield of about 0.055, and fluorescence lifetimes of 1.3–2.6 ns, were determined [64].

The above short review shows that the single absorption and emission spectra were measured for (−)-cytisine and its selected derivatives in solutions, but solvatochromic studies were not conducted for any of them. It is therefore difficult to predict the influence of solvent properties on the interactions between the newly synthesized in this study (−)-cytisine derivative and the solvent or a more complex environment. We decided to perform such a study for both *N*- propargylcytisine (**A**) and its target dimer (**B**).

To determine the effect of solvent polarity on the bands shift in the absorption spectra of *N*-propargylcytisine (**A**) and its dimer (**B**), their spectra were recorded in n-hexane, 1-chloro-n-butane, and acetonitrile as solvents differing significantly in polarity and nonspecifically interacting with the molecules of the compounds studied. Considering the presence of free electron pairs on the oxygen atom of the carbonyl group in the molecules of *N*-propargylcytisine (**A**) and its dimer (**B**), we used the solvents that can form donor-type hydrogen bonds, such as water and methanol, characterized by a polarity function like that of acetonitrile.

## 2. Results and Discussion

**Scheme 13:**
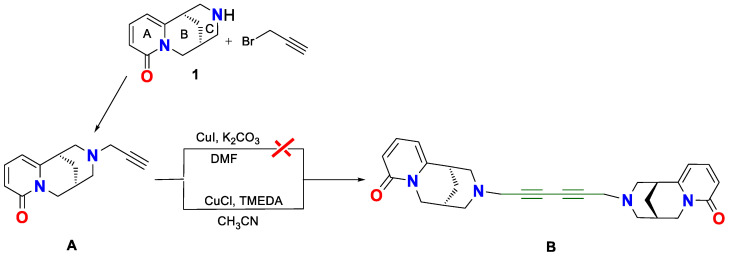
Synthesis of symmetric cytisine dimer via Glaser–Hay coupling reaction.

As the affinity of the synthesized cytisine derivatives **3** and **4** (Figure 1) to specific nACh receptor subtypes including a triple bond in their chain had been well-established, we pursued the synthesis of novel derivatives. Using *N*-propargylcytisine (**A**) as the key substrate and a coupling reaction strategy, we have designed a method for synthesizing the target dimer 1,6-dicytisinyl-hexa 2,4-diyne (**B,** Scheme 13).

Compound **A** was synthesized via direct alkylation of (−)-cytisine (**1**) with propargyl bromide (Scheme 13). In contrast to previously reported procedures [18,47], our method did not require a base catalyst. The resulting *N*-propargylcytisine (**A**) exhibited good solubility in various organic solvents, including dichloromethane, methanol, and acetonitrile, and remained stable at room temperature without detectable degradation.

Two methods for synthesizing *N*-propargylcytisine (**A**) have been reported earlier. One employed toluene as the solvent under reflux and a nitrogen atmosphere, affording a 60% yield. The final product was characterized by elemental analysis and a melting point, as well as molecular modeling to assess structure–activity relationships, indicating notable binding affinity (pKi = 7.00) for nAChRs [18]. The other method [47] used acetone at 50 °C for 6 h, affording the product as white crystals in 65% yield. The product was characterized by X-ray crystallography, IR, and ^1^H NMR spectroscopy [47]. The ^1^H NMR spectrum showed the signals assigned to cytisinyl and propargyl protons and with the CH_2_ group appearing as a doublet at 3.5 ppm and the methine proton as a triplet at 2.8 ppm. The IR spectrum exhibited the absorption bands characteristic of the C≡C bond at 2110 cm^−1^ and the lactam carbonyl at 1660 cm^−1^. However, detailed spectral assignments have not been reported.

**Figure 4:**
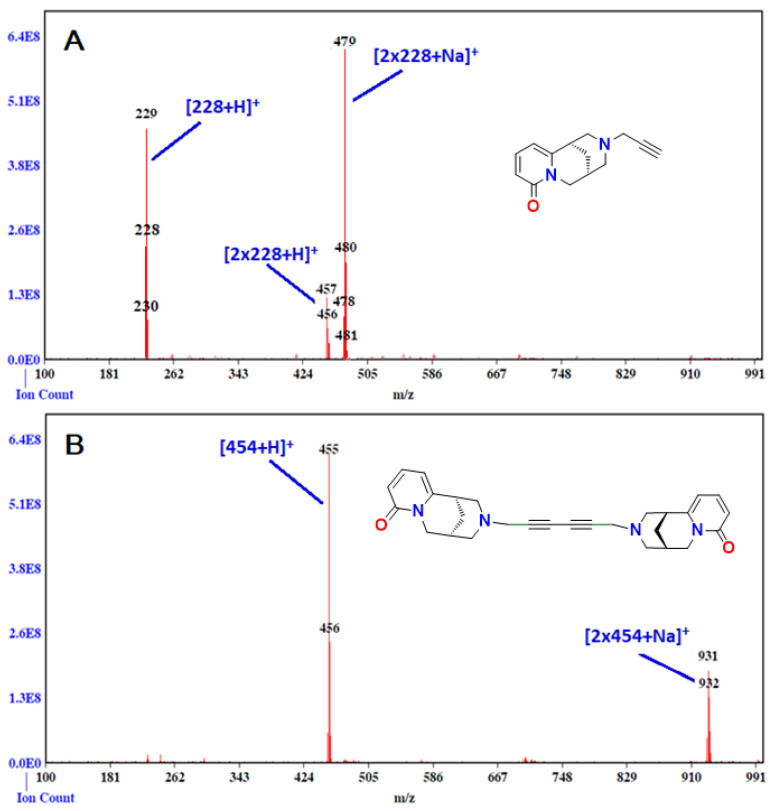
ESI mass spectrum of N-propargylcytisine (**A**), C_14_H_16_N_2_O; MW 228 and its dimer (**B**), C_28_H_30_N_4_O_2_, MW 454.57.

**Figure 5:**
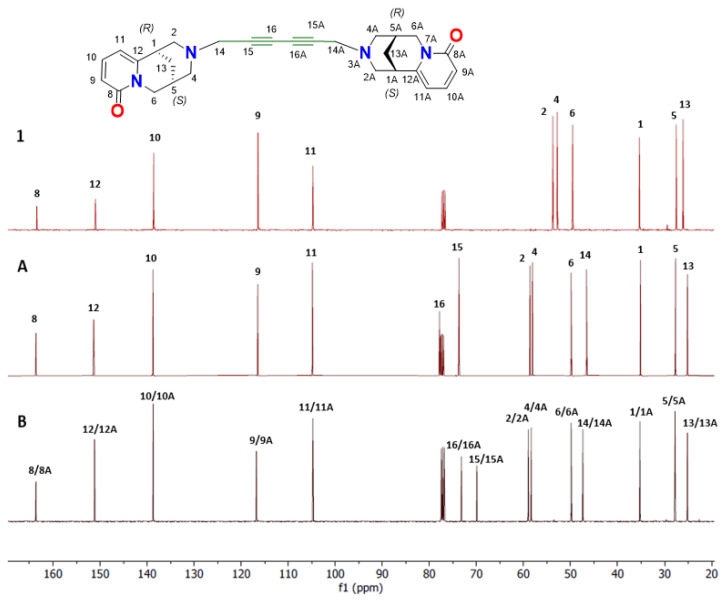
Comparison of chemical shifts of ^13^C-NMR spectra (CDCl_3_, 151 MHz) of (−)-cytisine (**1**, red line), *N*-propargyl-cytisine (**A**, maroon line), and its dimer (**B**, black line).

**Figure 6:**
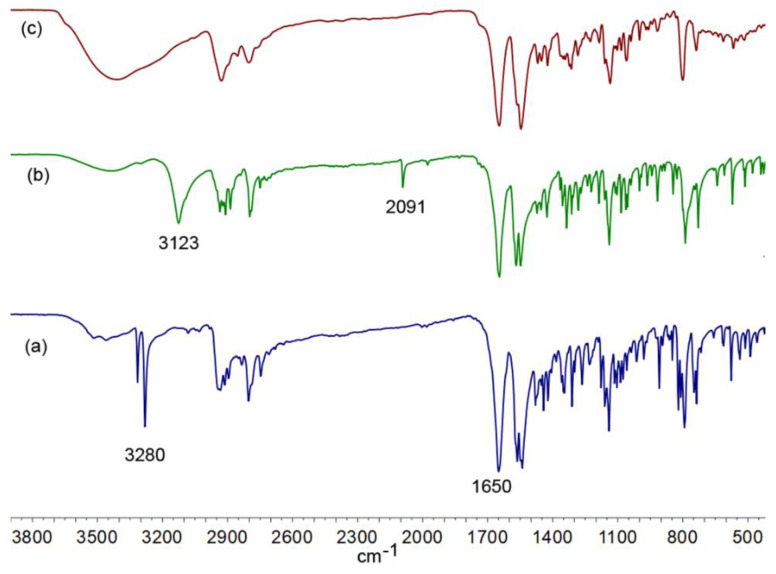
A comparison of the FT-IR spectra measured in KBr of (**a**) (−)-cytisine (**1**), (**b**) *N*-propargyl-cytisine (**A**), and (**c**) (−)-cytisine dimer (**B**).

In contrast, our method proceeded at room temperature in a mixture of dichloromethane and methanol (50:1, *v*/*v*) without the use of an external base, yielding compound **A** in the yield of 62%. The product identity was confirmed by ESI-MS (Figure 4), and it was fully characterized using ^13^C NMR (Figure 5, Table 2) and IR spectroscopy (Figure 6).

The synthesis of dimer **B** was first attempted under classical Glaser coupling conditions using compound **A**, CuI, and 0.5 equivalents of K_2_CO_3_ in DMF under an oxygen atmosphere at room temperature [34] (Scheme 13). However, ESI-MS analysis did not permit detection of the formation of the desired diyne-linked dimer. Therefore, we employed the Glaser–Hay protocol [37], utilizing CuCl and TMEDA in acetonitrile under an oxygen atmosphere. This time, ESI-MS analysis confirmed the formation of dimer **B**, with the molecular ion corresponding to C_28_H_30_N_4_O_2_ (m/z = 455, Figure 4). The isolated dimer exhibited a melting point of 117 °C, slightly higher than that of monomer **A** (108–110 °C). Notably, a previously reported cytisine-based dimer containing a single alkyne group displayed a substantially higher melting point (202–204 °C) [18].

Solvent screening revealed that acetonitrile was optimal for the Glaser–Hay coupling reaction. In contrast, reactions in DMF failed to yield the desired product, while THF led to significant by-product formation. Temperature optimization showed that the highest yield (70%) was obtained at 50 °C, whereas room temperature reactions were less efficient (65%) and required longer reaction times.

Another important parameter was the amount of copper catalyst. The catalyst loading of 0.1 equivalent of Cu(I)) was ineffective, probably due to competitive coordination between copper and the free amine group in the substrate. Increase in the catalyst loading to 0.5 equivalents of Cu(I) significantly improved the coupling [37]. Among the bases tested, TMEDA proved superior to triethylamine and triethanolamine [65], most probably because of its stronger and more selective chelation to the copper center, thereby promoting the formation of catalytically active Cu–alkynyl species.

The novel cytisine dimer **B** was thoroughly characterized by ESI-MS (Figure 4), ^13^C-NMR (Figure 5, Table 2) and IR spectroscopy (Figure 6). Although the detailed reaction mechanism remains to be fully elucidated, the process is hypothesized to proceed via a Cu(I)/Cu(III)/Cu(II)/Cu(I) redox cycle, with molecular oxygen acting as the oxidant to facilitate the coupling of terminal alkynes [66].

### Spectroscopic Analysis

The ESI-MS spectra (Figure 4) confirmed the presence of the desired compounds **A** and **B**, indicating that the target compounds were successfully obtained. The observed mass-to-charge ratios (m/z) suggest that neither compound **A** nor **B** coordinates metal ion in the 1:1 stoichiometry.

However, the spectra show that both compounds can readily coordinate sodium ion (Na^+^) in the 2:1 ligand-to-metal stoichiometry, forming dimeric species [2L+Na]^+^. Furthermore, both derivatives **A** and **B** exhibit a tendency to form dimers. Notably, compound **B** demonstrates a lower tendency for dimerization and sodium ion coordination compared with compound **A.** In the ESI-MS spectrum of compound **B**, the [L+H]^+^ ion appears as the dominant signal.

The ^13^C-NMR spectrum of cytisine dimer (**B**) was analyzed and compared with the spectra of (−)-cytisine (**1**) and *N*-propargylcytisine (**A**) (Figure 5, Table 2). A detailed comparison of the chemical shifts is provided in Table 2. Notably, analysis of the ^13^C-NMR spectra of cytisine (**1**) and *N*-propargylcytisine (**A**) reveals changes in the chemical environment of several carbons in ring *C*. In *N*-propargylcytisine (**A**), the resonances for signals assigned to C1, C2, C4, and C13 were shifted by +0.4, −4.5, −5.1, and +1.1 ppm, respectively, relative to their position in the spectrum of (−)-cytisine (**1**). The modest downfield shifts observed for C1 and C13 suggest slight electron-withdrawing effects or local structural changes caused by the propargyl group. In contrast, the significant upfield shifts at C2 and C4 (−4.5 and −5.1 ppm) indicate increased shielding, likely due to electron-donating interactions of the propargyl substituent. The chemical shifts in *N*-propargylcytisine (**A**) were further compared with those of the newly synthesized cytisine dimer (**B**). The observed differences are generally small, indicating that most carbon atoms in the dimer experience similar electronic environments. However, noticeable deviations begin at C14, which shows a shift of -0.7 ppm. More pronounced differences are observed at C15 and C16, where the signals shift upfield to lower frequency values (73.1 and 69.8 ppm) compared with those in the spectrum of compound **A** (77.8 and 73.1 ppm), as shown in Table 2. The chemical shift differences for C15 and C16 are 4.5 and 3.7 ppm, respectively.

These significant changes point to structural rearrangements upon dimerization, which affect the electron density around the acetylenic carbons. A similar trend has been reported in the literature, for diyne systems, for which substantial upfield shifts have been observed for acetylenic carbons [35,67]. In conclusion, the observed ^13^C NMR chemical shift variations reflect both the influence of the propargyl group on the electronic distribution within the cytisine framework and the structural changes associated with dimerization, particularly in regions surrounding the ring *C* and the acetylenic moiety.

The FT-IR spectra of (−)-cytisine (**1**), *N*-propargyl-cytisine (**A**), and the synthesized (−)-cytisine dimer (**B**) measured in KBr are presented in Figure 6. The spectrum of (−)-cytisine (**1**) exhibits the absorption band characteristics of its functional groups. A broad band at 3280 cm^−1^ is attributed to N–H stretching vibrations, indicating the amine group presence. The prominent bands at 1650 cm^−1^ and 1563 cm^−1^ correspond to the -C=O and -C=C stretching vibration of the lactam moiety of a quasi-aromatic ring. The spectrum of *N*-propargyl-cytisine (**A**), presented in [47], shows distinctive features related to the introduced propargyl group. The C≡C stretching vibration appears at 2091 cm^−1^, while the broad absorption band at 3123 cm^−1^ is assigned to the terminal ≡C–H stretching. The C=O and C=C stretching vibrations are observed at 1650 cm^−1^ and 1563 cm^−1^, respectively. Notably, the N–H stretching band present in (−)-cytisine is absent in the spectrum of compound **A**, confirming the propargylation of the amine nitrogen. A comparison of this spectrum with that of the synthesized dimer (**B**) reveals further structural transformations. A broad band in the range of 3200–3600 cm^−1^, observed in the spectrum of compound **B** recorded in KBr, is probably a result of the environmental moisture. The absence of the ≡C–H stretching band indicates consumption of the terminal alkyne. In these three spectra, the bands corresponding to C=O (1650 cm^−1^) and C=C (1563 cm^−1^) stretching vibrations, are consistently present, confirming the retention of these functional groups in the derivatives.

**Figure 7:**
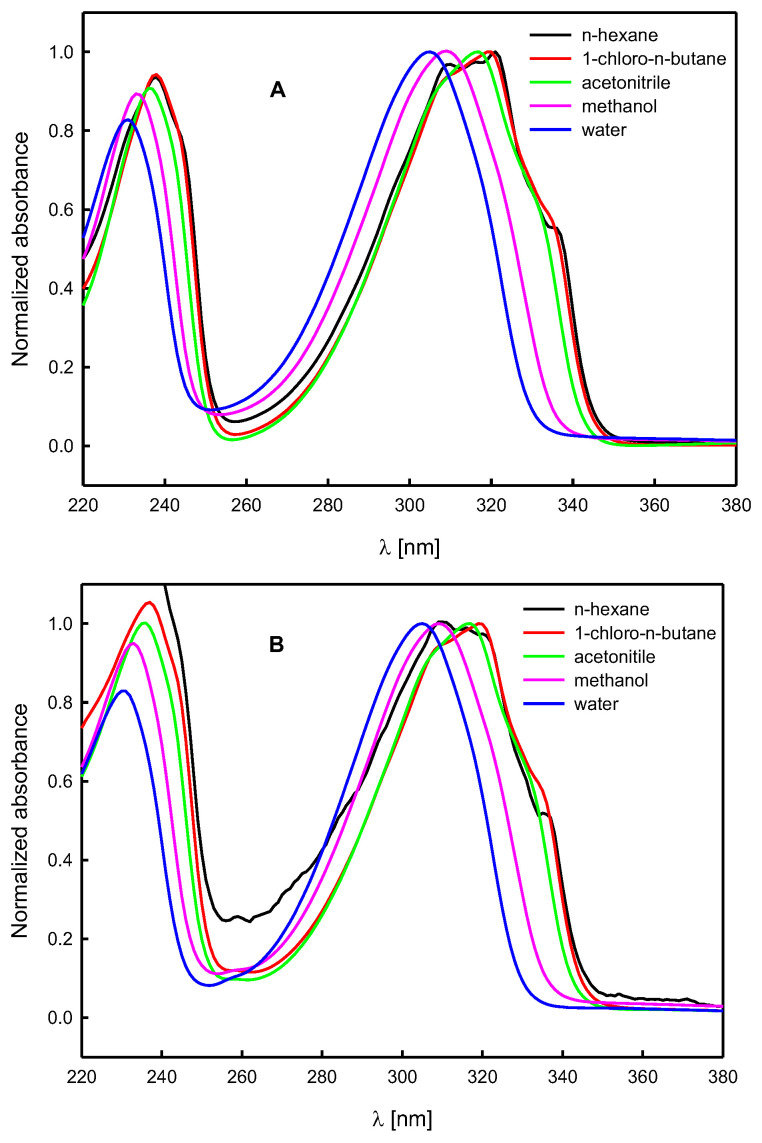
Normalized UV-VIS absorption spectra of *N*-propargylcytisine (**A**) and (−)-cytisine dimer (**B**) in solvents of different properties.

The UV-VIS absorption spectra of *N*-propargylcytisine (**A**) and its dimer (**B**) were recorded in a few solvents of different polarities, including two protic ones capable of hydrogen bonding (Table 1 and Table 3). For both compounds studied, a hypsochromic shift in the maximum of the long wavelength band in the absorption spectrum, λ^max^, was observed with solvent polarity function, f(ε,n^2^), increasing (Figure 7, Table 3). The shifts in the spectra recorded in methanol and water were significantly greater than in the other solvents. As these solvents have polarities like that of acetonitrile, it indicates that the solute–solvent complexes form through intermolecular hydrogen bonding. Moreover, the shift in the band maximum in the spectrum recorded in water was greater than that observed for methanol. It should be added that water is characterized by a higher value of the Kamlet–Taft’s solvatochromic parameter related to hydrogen-bond donating ability, α (Table 1).

**Figure 8:**
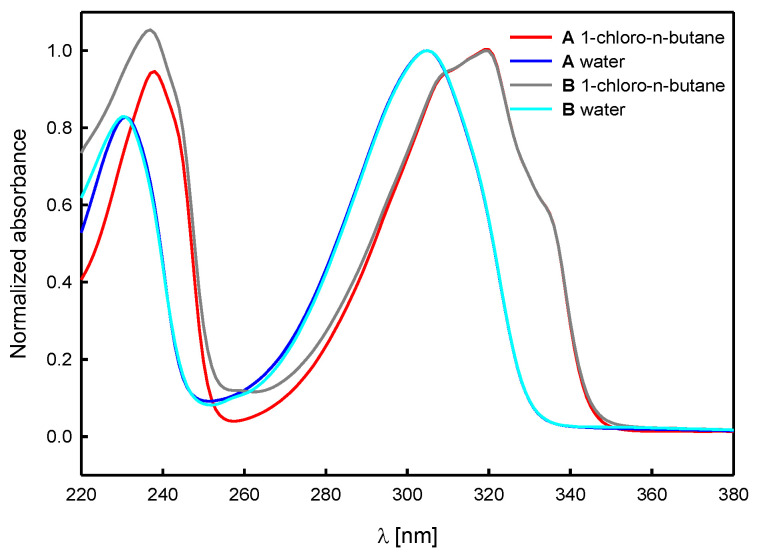
A comparison of UV-VIS absorption spectra (normalized) of *N*-propargylcytisine (**A**) and (−)-cytisine dimer (**B**) in selected solvents.

The shape and position of the bands in the absorption spectra of compounds **A** and **B** do not differ significantly from each other (Figure 8). Moreover, they are like those in the absorption spectra of (−)-cytisine [59,60,62], and its derivatives [61,62,64]. They are also typical of some quinolizidine alkaloids containing α-pyridone moiety [68,69,70,71,72]. The value of the molar absorption coefficient at the maximum of the long wavelength band, ε^max^, for both compounds studied, **A** and **B,** increases in solvents of higher polarity (Table 3). The values of ε^max^ for *N*-propargylcytisine (**A**) are similar to those obtained for (−)-cytisine [59,60] and sophazrine [69].

Interestingly, one order of magnitude lower values of molar absorption coefficient have been reported for 3-amino derivatives of *N*-methylcytisine [64] and the anagyrine derivative [71]. The newly synthesized in this study (−)-cytisine dimer (**B**) is characterized by ε^max^ values of 1.7–1.9 times higher than that of *N*-propargylcytisine (Table 3).

**Figure 9:**
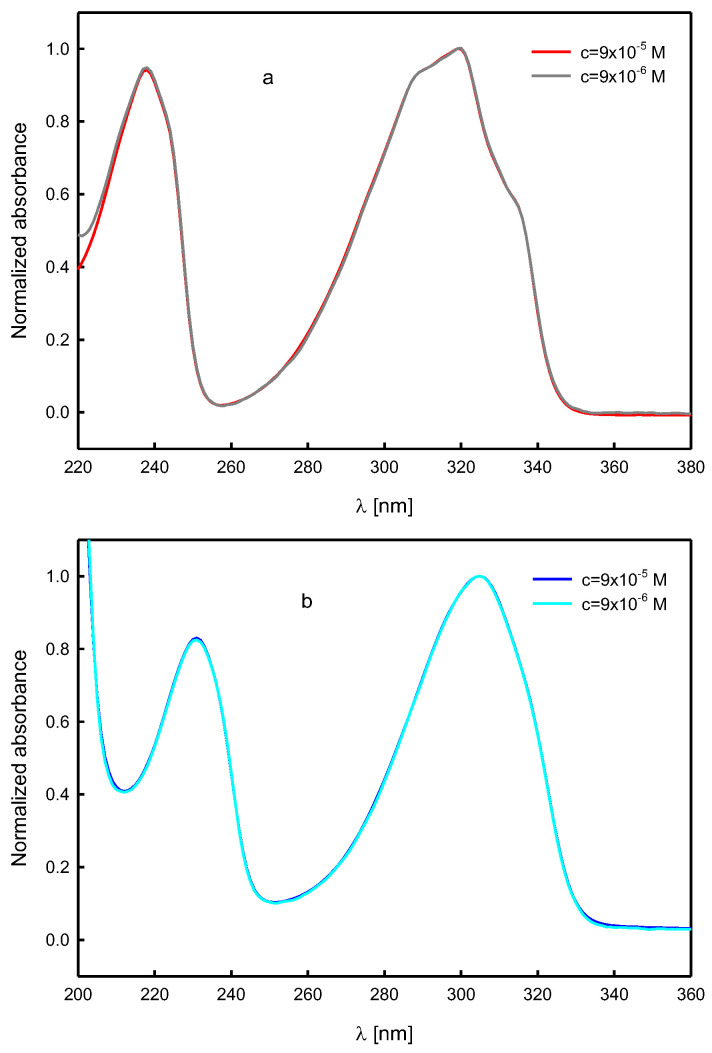
Effect of the solute concentration on the UV-VIS absorption spectra of *N*-propargylcytisine (**A**) in 1-chloro-n-butane (**a**) and water (**b**) (normalized spectra).

To exclude aggregation of compound molecules in the solution, the UV-VIS absorption spectra of **A** and **B** in all solvents used in the study were measured for two concentrations of the solute, c~10^−5^ M and c~10^−6^ M. There was no significant effect of the solute concentration on the results obtained (Figure 9).

## 3. Materials and Methods

### 3.1. General

Reagents: propargyl bromide (81831, ~80% solution in toluene) and TMEDA were purchased from Fluka/Fisher Scientific, Loughborough, UK. All other commercially available reagents and solvents were obtained from POCH, Gliwice, Poland, and used without further purification.

*Spectroscopic measureme.nts: ESI mass spectra* were obtained on a Waters/Micromass (Manchester, UK) ZQ mass spectrometer (MassLynx V4.0). The samples were prepared in methanol or in methanol–water solution. The concentration of the analyzed samples was 3.6 d 10^−5^ M (1:1 ratio), which is typical of ESI, unless indicated otherwise.

The *he FT-IR spectra* of each compound were recorded in KBr tablets on an IFS 113 v FT-IR spectrophotometer (Bruker, Karlsruhe, Germany) equipped with a DTGS detector; wavenumber (cm^–1^) resolution of 2 cm^–1^; NSS = 125; description: w = weak, m = medium, s = strong, br = broad. The Happ-Genzel apodization function was used.

*The NMR spectra* of the products were recorded on a Bruker AvanceNEO 600 (Bruker, Karlsruhe, Germany). The ^1^H NMR measurements were carried out at the operating frequency of 600.1 MHz and the ^13^C NMR at 151.2 MHz at 293.0 K. No window function or zero filling was used. The ^1^H NMR spectra were reported in chemical shifts downfield from CDCl_3_ δ 7.26 ppm using the respective residual solvent peak as internal standard. Line broadening parameters were 0.5 or 1.0 Hz, while the error of chemical shift value was 0.1 ppm. The ^1^H NMR spectra are described as follows: chemical shift (δ, ppm) and integration and multiplicity (s = singlet, d = doublet, dd = doublet of doublets, m = multiplet). The ^13^C NMR spectra were reported in chemical shifts downfield from the respective residual solvent peak as internal standard (CDCl_3_ δ 77.16 ppm). Line broadening parameters were 0.5 or 1.0 Hz, while the error of chemical shift value was 0.1 ppm.

*The UV-VIS spectra* of *N-propargylcytisine (**A**) and its dimer (**B**)* were recorded on a Jasco V-650 spectrophotometer (Jasco, Hachioji, Japan) at room temperature. The solvents: n-hexane (Sigma-Aldrich, ≥97%, Poznań, Poland), 1-chloro-*n*-butane (≥99.8%, POCH, Poznań, Poland), and acetonitrile (chemsolve, ≥99.9%, Poznań, Poland) were additionally dried over the molecular sieves A3; methanol (chemsolve, ≥99.85%, Poznań, Poland) and water (for HPLC, POCH, Poznań, Poland) was used as received.

### 3.2. Synthesis of (−)-Cytisine Derivatives A and B

***Synthesis of N-propargylcytisine*** (**A**, Scheme 13): (−)-cytisine (**1**, 0.986 g, 5.17 mmol, 1 eq.) and propargyl bromide (1.078 mL, 12.82 mmol, 2.48 eq) were dissolved in a mixture of CH_2_Cl_2_/MeOH, 100:2. The reaction mixture was stirred at room temperature in a closed container. A white precipitate began to form within 1 h. The reaction was monitored by TLC and after completion (~48 h), the solvent was removed under reduced pressure. Further purification was achieved by column chromatography using CH_2_Cl_2_/MeOH 9:1, yielding *N*-propargyl-cytisine as a creamy solid (0.735 g, 62%). *N*-propargyl-cytisine (**A**) C_14_H_16_N_2_O MW 228; m.p. 108–110 °C (lit.113–114 °C) [43]; anal. calcd for C, 73.66; H, 7.06; N, 12.27; found C, 73.87; H, 7.15; N, 12.19; ^1^H NMR (CDCl_3_, 600 MHz, Supplementary Materials), δ in ppm, integration and multiplicity: 7.23, (1H, dd); 6.39 (1H, dd); 5.94 (1H, dd); 3.99 (1H, d); 3.18 (2H, m); 2.94 (1H, m); 2.77 (1H, m); 2.70 (1H, m), 2.61 (2H, m), 2.44 (1H, m), 2.18 (1H, s); 1.78 (2H, m). ^13^C NMR (CDCl_3_, 151 MHz, Table 2, Figure 5)*;* IR: 2091 cm^−1^ (of terminal -C≡C-H), 1650 cm^−1^ (ν C=O), 1563 cm^−1^ (ν C=C) is given in Figure 6 and the UV-VIS spectra in Figure 7 and Figure 8.

***Synthesis of (−)-cytisine dimer*** (**B**, Scheme 13): *N*-propargylcytisine (**A**, 0.456 g, 2 mmol), TMEDA (MW 116; d = 0.77; 0.148 mL, 1 mmol) and CuCl (0.098 g, 1 mmol) were added to round-bottom flask and dissolved in acetonitrile (30 mL) under an air atmosphere. The reaction mixture was vigorously stirred at 50 °C for 12 h. Initially, the mixture appeared light blue, but after 10 min, it turned dark blue. The progress of reaction was monitored by TLC. Upon completion, acetonitrile was evaporated under reduced pressure. The reaction mixture was then treated with DCM and washed with sat. aq. EDTA solution (3 × 35 mL). The organic layer was separated, dried over MgSO_4_, and concentrated under reduced pressure. The resulting crude product was purified by flash column chromatography (SiO_2_) using CH_2_Cl_2_/MeOH 19:1, yielding product **B** as a creamy solid. (0.32 g, 70%). m.p. 117 °C; *m*/*z*: 455 [M + H], Anal. Calcd for C_28_H_30_N_4_O_2_: C, 73.98; H, 6.65; N, 12.33; found C, 73.83; H, 6.78; N, 12.42. ^1^H NMR (CDCl_3_, 600 MHz, Supplementary Materials), δ in ppm, integration and multiplicity: 7.23, (1H, dd); 6.39 (1H, dd); 5.94 (1H, dd); 3.99 (1H, d); 3.86 (2H, m); 3.25 (2H, m), 2.95 (1H, m); 2.79 (1H, m); 2.64 (3H, m), 2.45 (1H, m), 1.79 (2H, m). ^13^C NMR (CDCl_3_, 600 MHz, Table 2, Figure 5); IR (FT-IR, Figure 6): the absence of the band of terminal ν -C≡C-H, 1650 cm^−1^ (ν C=O), 1563 cm^−1^ (ν C=C) is shown in Figure 6 and the UV-VIS spectra in Figure 7 and Figure 8.

## 4. Conclusions

Taking into account the well-established affinity of cytisine-based dimer to specific nicotinic acetylcholine receptors (nAChRs), particularly those incorporating triple bonds, a new dimeric derivative (**B**) was synthesized via Glaser–Hay coupling of *N*-propargylcytisine (**A**). The precursor A was obtained through direct alkylation of (–)-cytisine (**1**) with propargyl bromide under mild, base-free conditions, affording a good yield and offering advantages over previous protocols in terms of operational simplicity, solubility, and stability.

It is worth noting that the initial attempts to synthesize the diyne-linked dimer **B** using classical Glaser conditions proved unsuccessful; however, the Glaser–Hay method, employing CuCl/TMEDA in acetonitrile under an oxygen atmosphere at 50 °C, enabled efficient coupling, yielding compound **B** in a satisfactory yield (70%). Spectroscopic characterization by ESI-MS, ^1^H and ^13^C NMR, and IR spectroscopy confirmed the successful formation and integrity of the target molecule. ESI-MS revealed that both A and B formed [2L+Na]^+^ adducts, though **B** exhibited a lower tendency for dimerization and sodium ion coordination. The ^13^C NMR spectra showed notable chemical shift changes upon propargylation and dimerization, particularly at the acetylenic carbons, in agreement with diyne formation. Likewise, the IR spectroscopy indicated the disappearance of the terminal ≡C–H vibration and the emergence of a diyne C≡C band, along with a new amide C=O stretching band. Further insights from the UV–VIS spectroscopy demonstrated solvatochromic effects for compounds **A** and **B**, with significant hypsochromic shifts in protic solvents, suggesting solute–solvent hydrogen bonding interactions. Molar absorption coefficient increased with solvent polarity, and the diyne-linked dimer **B** displayed values of approximately 1.7–1.9 times higher than those of the derivative **A**. Additionally, no evidence of aggregation was observed at the concentrations tested, supporting the monomeric behavior of both compounds in solution.

## Figures and Tables

**Table 1:** Solvent polarity and hydrogen-bond formation ability parameters.

Solvent	f(ε,n^2^)	ε *^a^*	n *^a^*	α *^a^*	β *^a^*
n-hexane	−0.0003	1.88	1.372	0.00	0.00
1-chloro-n-butane	0.209	7.39	1.400	0.00	0.00
HMPA	0.261	29.30	1.457	0.00	1.00
acetonitrile	0.306	35.94	1.341	0.19	0.40
methanol	0.309	32.66	1.328	0.98	0.66
water	0.320	78.00	1.333	1.17	0.47
HFIP	0.308	16.62	1.277	1.96	0.00

f(ε,n^2^) = ((ε − 1)/(2ε + 1)) − ((n^2^ − 1)/(2n^2^ + 1))—solvent polarity function, n—refractive index, ε—relative permittivity, α—Kamlet–Taft’s solvatochromic parameter related to hydrogen-bond donating ability, β—Kamlet–Taft’s solvatochromic parameter related to hydrogen-bond accepting ability, ^a^ from Ref. [57].

**Table 2:** Summary of the chemical shifts in carbon atoms in the measured ^13^C NMR (δ [ppm]) of cytisine (**1**) and **A** and **B** in CDCl_3_ (151 MHz, Bruker AvanceNEO 600).

C Atoms	1	Δδ (1-A)	A	B	Δδ (A-B)
1	35.6	0.4	35.2	35.2 (1/1A)	0
2	54.0	−4.6	58.6 ^a^	58.9 (2/2A) ^a^	−0.3
4	53.0	−5.1	58.1 ^a^	58.3 (4/4A) ^a^	−0.2
5	27.7	0	27.7	27.7 (5/5A)	0.1
6	49.7	0.1	49.8	49.8 (6/6A)	0
8	163.6	0.1	163.5	163.5 (8/8A)	0
9	116.6	0.2	116.7	116.7 (9/9A)	0
10	138.8	0.3	138.5	138.6 (10/10A)	0.1
11	104.9	0.4	104.5	104.6 (11/11A)	−0.1
12	151.1	0.1	151.2	151.0 (12/12A)	0.2
13	26.3	1.1	25.2	25.1 (13/13A)	−0.1
14			46.6	47.3 (14/14A)	−0.7
15			77.8	73.1 (15/15A)	4.5
16			73.5	69.8 (16/16A)	3.7

a—interchangeable signals.

**Table 3:** Effects of the solvent properties on the UV-VIS absorption spectra of (−)-cytisine (**1**), *N*-propargylcytisine (**A**), and (−)-cytisine dimer (**B**).

Solvent	(1)		A		B	
	λ^max^ [nm]	ε^max^ [M^−1^cm^−1^]	λ^max^ [nm]	ε^max^ [M^−1^cm^−1^]	λ^max^ [nm]	ε^max^ [M^−1^cm^−1^]
n-hexane	322 ^a^	6070 ^a^	321	7100	321	-
1-chloro- n-butane	320	6240	319	6950	319	12,100
acetonitrile	318 ^b^	7240 ^b^	317	7250	317	13,300
methanol	310	7760	309	8300	309	15,800
water	307	8090	305	8200	305	13,800

λ^max^—long wavelength band maximum, ε^max^—molar absorption coefficient at the maximum of the long wavelength band, ^a^ after Ref. [59], ^b^ after Ref. [60].

## Data Availability

Dataset available on request from the authors.

## Supplementary Materials

The following supporting information can be downloaded at: https://www.mdpi.com/article/10.3390/molecules30193955/s1, Scheme S1: Synthesis of symmetric cytisine dimer via Glaser-Hay coupling reaction; Figure S1: The chemical shifts of 1H NMR (600 MHz) and 13C-NMR spectra (151 MHz) in CDCl3 of N-propargyl-cytisine (**A**); Figure S2: The chemical shifts of 1H NMR (600 MHz) and 13C-NMR spectra (151 MHz) in CDCl3 of dimer propargylcytisine (**B**).
