# Supplementary material for: Copper-Mediated Homocoupling of N-propargylcytisine—Synthesis and Spectral Characterization of Novel Cytisine-Based Diyne Dimer
Source: Molecules. 2025 Oct 1;30(19):3955. doi: 10.3390/molecules30193955 (PMC12526314; doi:10.3390/molecules30193955)
Supplement: Supplementary file 1 [file molecules-30-03955-s001.zip › molecules-3819708-supplementary.pdf]

Supplementary data

Copper-Mediated Homocoupling of *N*-propargylcytisine – Synthesis and Spectral Characterization of Novel Cytisine-Based Diyne Dimer

Anna K. Przybył [1]*, Adam Huczyński [1] and Ewa Krystkowiak [2]

[1] Department of Medical Chemistry, Faculty of Chemistry, Adam Mickiewicz University, Uniwersytetu Poznańskiego 8, 61-614, Poznań, Poland; anna.przybyl@amu.edu.pl (A.K. P), adhucz@amu.edu.pl (A.H)

[2] Department of Spectroscopy and Magnetism, Faculty of Chemistry, Adam Mickiewicz University, Uniwersytetu Poznańskiego 8, 61-614, Poznań, Poland; ewakryst@amu.edu.pl (E. K)

* Correspondence: anna.przybyl@amu.edu.pl (A.K.P)

Abstract: Cytisine, a naturally occurring alkaloid and partial agonist of nicotinic acetylcholine receptors (nAChRs), has long been used as a smoking cessation aid and serves as the pharmacophore for varenicline. Recent research has expanded its therapeutic scope to neurodegenerative and neurological disorders, motivating the development of new cytisine derivatives. Among these, *N*-propargylcytisine combines the biological activity of the parent compound with the synthetic versatility of the terminal alkyne group. Herein, we report the synthesis and characterization of *N*-propargylcytisine and its symmetrical dimer linked through 1,3-diyne moiety obtained via a copper-mediated Glaser–Hay oxidative coupling. The products were analyzed by NMR, FT-IR, and mass spectrometry, confirming the introduction of the propargyl moiety and the formation of the diyne bridge. Solvatochromic study of both compounds were performed using UV-VIS absorption spectroscopy in solvents of varying polarity, including protic solvents capable of hydrogen bonding. The 1,3-diyne motif, commonly found in bioactive natural products, endows the resulting dimer with potential for further derivatization and biological evaluation. This study demonstrates the utility of the Glaser–Hay reaction in the functionalization of alkaloid scaffolds and highlights the prospects of *N*-propargylcytisine derivatives in drug discovery targeting the central nervous system.

Keywords: *N*-heterocycles, alkaloids, (-)-cytisine derivatives, *N*-propargylcytisine, Glaser-Hay coupling, 1,3-diynes, alkaloid functionalization, UV-Vis absorption spectroscopy.

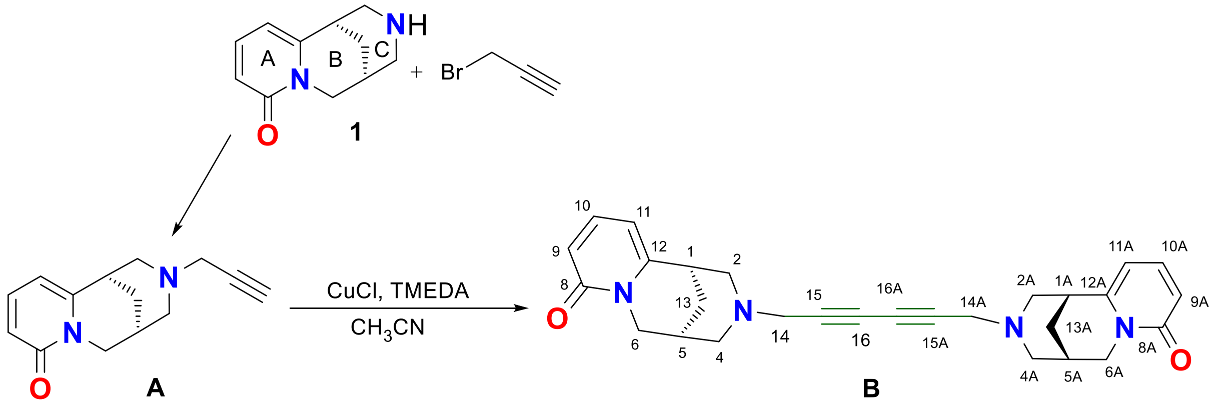

Scheme S1. Synthesis of symmetric cytisine dimer via Glaser-Hay coupling reaction.

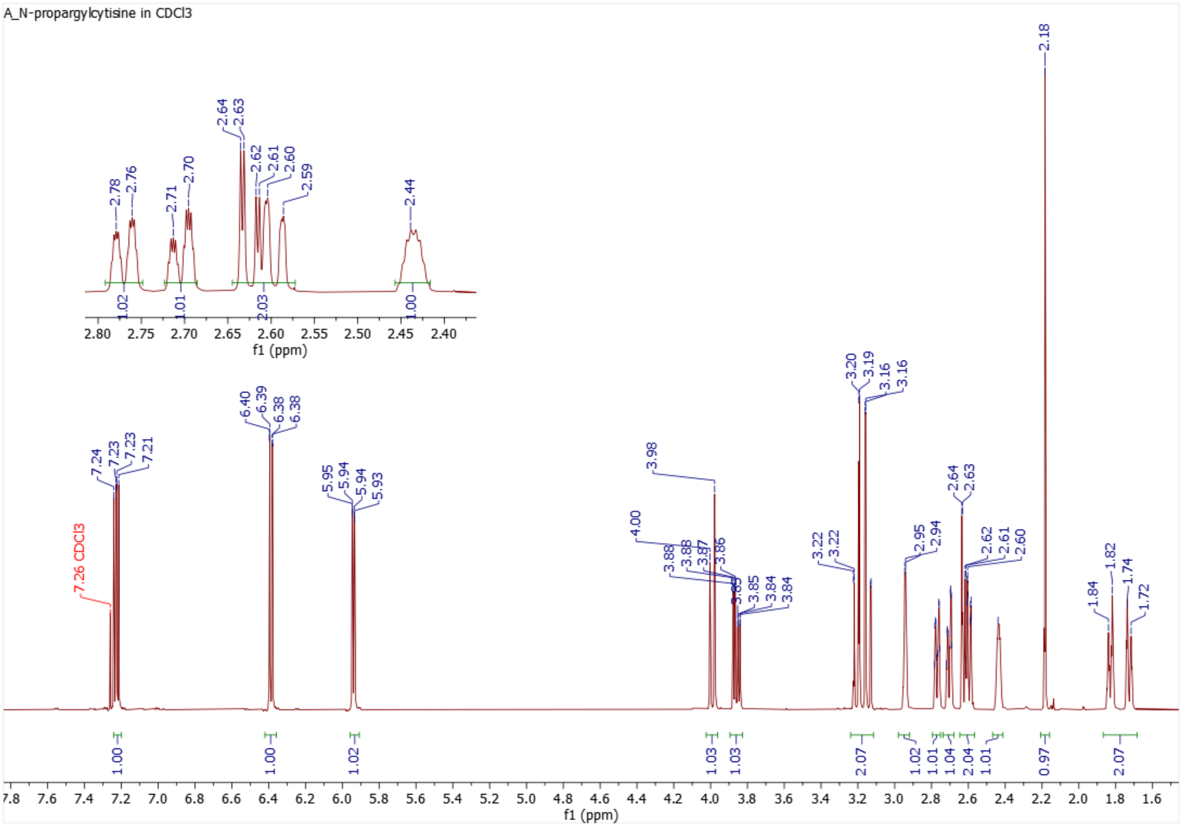

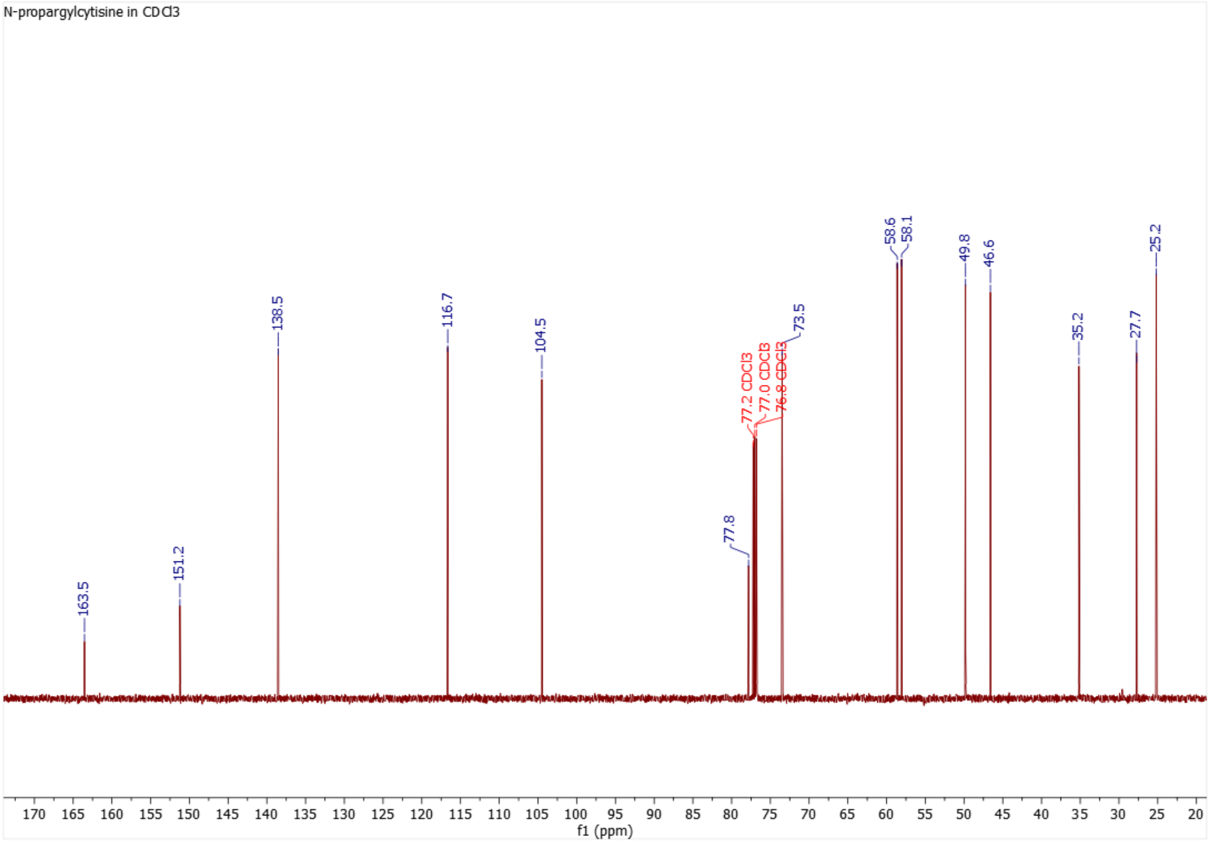

Figure S1. The chemical shifts of ^{1}H NMR (600 MHz) and ^{13}C-NMR spectra (151 MHz) in $CDCl_3$ of *N*-propargyl-cytisine (**A**).

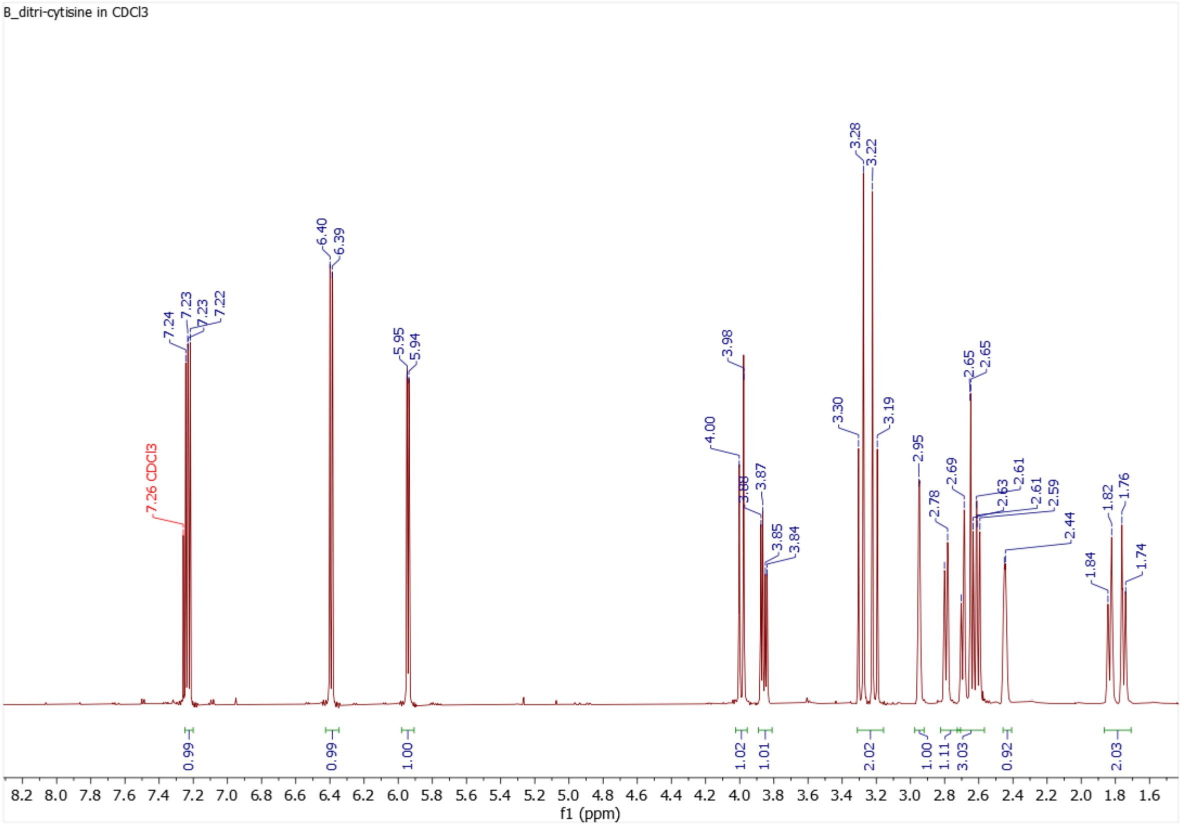

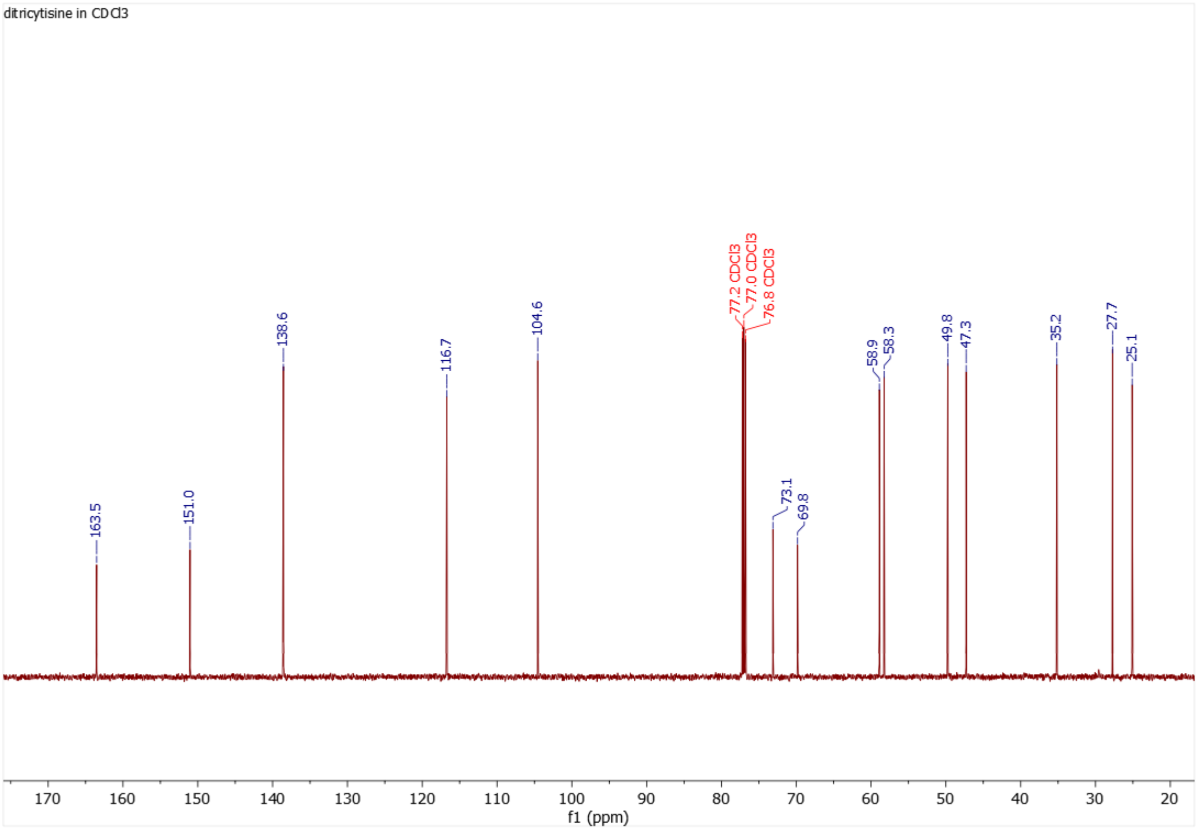

Figure S2. The chemical shifts of ^{1}H NMR (600 MHz) and ^{13}C-NMR spectra (151 MHz) in $CDCl_3$ of dimer propargylcytisine (**B**).

1. Experimental

The NMR spectra of the products were recorded on a Bruker AvanceNEO 600. The ^{1}H NMR measurements were carried out at the operating frequency 600.1 MHz, while the ^{13}C NMR at 151.2 MHz at 293.0 K. No window function or zero filling was used. ^{1}H NMR spectra are reported in chemical shifts downfield from $CDCl_3$ δ 7.26 ppm using the respective residual solvent peak as internal standard. Line broadening parameters were 0.5 or 1.0 Hz, while the error of chemical shift value was 0.1 ppm. ^{1}H NMR spectra are described as follows: chemical shift (δ, ppm), integration and multiplicity (s = singlet, d = doublet, dd = doublet of doublets, m = multiplet). ^{13}C NMR spectra are reported in chemical shifts downfield from the respective residual solvent peak as internal standard ($CDCl_3$ δ 77.16 ppm). Line broadening parameters were 0.5 or 1.0 Hz, while the error of chemical shift value was 0.1 ppm.

3.2. Synthesis of (−)-cytisine derivatives **A** and **B**.

Synthesis of N-propargylcytisine (**A**, Scheme 13): (-)-cytisine (**1**, 0.986 g, 5.17 mmol, 1 eq.) and propargyl bromide (1.078 mL, 12.82 mmol, 2.48 eq) were dissolved in a mixture of CH_2Cl_2/MeOH, 100:2. The reaction mixture was stirred at room temperature in a closed container. A white precipitate began to form within 1 hour. The reaction was monitored by TLC and after completion (~48 h), the solvent was removed under reduced pressure. Further purification was achieved by column chromatography using CH_2Cl_2/MeOH 9:1, yielding *N*-propargyl-cytisine as a creamy solid (0.735 g, 62%). *N*-propargyl-cytisine (**A**) $C_{14}H_{16}N_2O$ MW 228; m.p. 108-110 ℃ (lit.113-114 ℃) [43]; Anal. Calcd for: C, 73.66; H, 7.06; N, 12.27; Found C, 73.87; H, 7.15; N, 12.19; ^{1}H NMR ($CDCl_3$, 600 MHz, supplementary data), δ in ppm, integration and multiplicity: 7.23, (1H, dd); 6.39 (1H, dd); 5.94 (1H, dd); 3.99 (1H, d); 3.18 (2H, m); 2.94 (1H, m); 2.77 (1H, m); 2.70 (1H, m), 2.61 (2H, m), 2.44 (1H, m), 2.18 (1H, s); 1.78 (2H, m). ^{13}C NMR ($CDCl_3$, 151 MHz, Table 2, Fig. 5 in the main text.); IR: 2091 cm^{-1} (of terminal -C≡C-H), 1650 cm^{-1} (C=O), 1563 cm^{-1} (ν C=C) is given in Figure 6 in the main text. The UV-VIS spectra in Figure 7 and 8 in the main text.

Synthesis of (-)-cytisine dimer (**B**, Scheme 13): *N*-propargylcytisine (**A**, 0.456 g, 2 mmol), TMEDA (MW 116; d=0.77; 0.148 mL, 1 mmol) and CuCl (0.098g, 1 mmol) were added to round-bottom flask and dissolved in acetonitrile (30 mL) under an air atmosphere. The reaction mixture was vigorously stirred at 50℃ for 12 hours. Initially, the mixture appeared light blue, but after 10 min. it turn dark blue. The progress of reaction was monitored by TLC. Upon completion, acetonitrile was evaporated under reduced pressure. The reaction mixture was then treated with DCM and washed with sat. aq. EDTA solution (3x35 mL). The organic layer was separated, dried over $MgSO_4$, and concentrated under reduced pressure. The resulting crude product was purified by flash column chromatography (SiO_2) using CH_2Cl_2/MeOH 19:1, yielding product **B** as a creamy solid. (0.32 g, 70 %). m.p. 117 ℃; *m/z*: 455 [M + H], Anal. Calcd for: $C_{28}H_{30}N_4O_2$: C, 73.98; H, 6.65; N, 12.33; Found: C, 73.83; H, 6.78; N, 12.42. ^{1}H NMR ($CDCl_3$, 600 MHz, supplementary data), δ in ppm, integration and multiplicity: 7.23, (1H, dd); 6.39 (1H, dd); 5.94 (1H, dd); 3.99 (1H, d); 3.86 (2H, m); 3.25 (2H, m), 2.95 (1H, m); 2.79 (1H, m); 2.64 (3H, m), 2.45 (1H, m), 1.79 (2H, m). ^{13}C NMR ($CDCl_3$, 600 MHz, Table 2, Fig. 5 in the main text.); IR (FT-IR, Fig. 6): the absence of the band of terminal -C≡C-H, 1650 cm^{-1} C=O), 1563 cm^{-1} (ν C=C) in Figure 6 in the main text. The UV-VIS spectra in Figure 7 and 8 in the main text.
